# Supplementary material for: Socioeconomic determinants of cancer screening utilisation in Latin America: A systematic review
Source: PLoS One. 2019 Nov 25;14(11):e0225667. doi: 10.1371/journal.pone.0225667 (PMC6876872; doi:10.1371/journal.pone.0225667)
Supplement: S1 Table — (DOCX) [file pone.0225667.s002.docx]

**S1 Table. Data extraction sheet headings**

| **REF** | **Author/ Year** | **Title** | **Objectives** | **Study Design** | **Study Design and/or Activities (what are they doing)** | **Primary Outcome** | **Secondary Outcome** | **Location of Study** | **Year of Study** | **Population of Interest** | **Results detail (relation between screening use and dependent variables)** | **Author's Recommendation** | **Limitations of the study** | **III.d What are the behavioral determinants for underuse of preventive health services?** |
| --- | --- | --- | --- | --- | --- | --- | --- | --- | --- | --- | --- | --- | --- | --- |
|  |  |  |  |  |  |  |  |  |  |  |  |  |  |  |
