# Supplementary material for: Socioeconomic determinants of cancer screening utilisation in Latin America: A systematic review
Source: PLoS One. 2019 Nov 25;14(11):e0225667. doi: 10.1371/journal.pone.0225667 (PMC6876872; doi:10.1371/journal.pone.0225667)
Supplement: Attachment — Submitted filename: renamed_6b233.docx [file pone.0225667.s005.docx]

PONE-D-19-18831

Socioeconomic predictors for low use of cancer screening in Latin America: a systematic review

The article is a narrative review of studies reporting on socioeconomic determinants for access to screening programs for three cancer sites between 2009 and 2018. Only 24 studies on the early detection of breast and cervical cancer were eligible for this analysis, but none for colorectal cancer. Therefore, the title could be changed to refer to screening for the early detection of breast and cervical cancer in women.

Abstract: The Result section should report on the effects of socioeconomic factors, separately for cervical and breast cancer.

Methods: The authors mention that information on the characteristics of the screening programs was frequently limited. However, the type of the program is pertinent for the analysis of socioeconomic factors. With regard to cancer (in particular cervical cancer) screening, it should be clarified, if the screening program was a research or pilot project with special aim to implement screening in a defined population such as a rural district (all socio-economic classes) or in special groups (indigenous women of low socio-economic status) or whether it is a general (nation-wide) free health-care program (for all socio-economic groups). Only the last type of screening programs allows an unbiased analysis of the participation by socio-economic status (SES), because all other screening programs are designed for selected subpopulations. If this information is limited, the analysis could be in two steps: for the studies selected and for those with sufficient information to be classified as a general population-based screening program.

Methods:

Table 1 indicates, with regard to cancer control, “Latin America” is a fractionated continent, but I have problems to understand this table aimed to show the search strategy. For example, the information on screening services and SES is lacking for several countries, such as Paraguay or Uruguay. A figure might be better. Most articles are from Brazil, and, for example, Argentina conducted an important pilot project to implement cervical cancer screening in a rural region. There was, for example, no report from Uruguay, classified with a “very high” Human Development Index and running a well-organized cancer registry.

Table 2 shows the search terms, this may be sufficient to delineate the search concept.

Results:

Because of differences in funding (higher for cervical than for breast cancer in Latin America) and in cancer risks by socio-economic status (SES) (higher risks for developing cervical cancer in women with low SES and higher risks for developing breast cancer in women with high SES), the analysis should be performed separately for these two cancer sites and presented in subtables.

Cervical cancer is of special concern in Latin America. Screening for the early detection of breast and colorectal cancer is less well organized. Cervical cancer screening is not just one step, but a stepwise procedure (HPV screening, if positive CIN screening). This should be considered when presenting the studies.

Challenges for cancer control in Latin America have been subject to comprehensive evaluations. Major problem is disparity in health care between metropolitan und rural regions. Therefore, the studies should be categorized by region (nation-wide, urban, rural).

A table showing the results by country and cancer site would be informative as first table of the Result section. The article should describe the characteristics of the region (country, nationwide or state or department), method (e.g. mammography) and target population more precisely in Table 3, separately for breast and cervical cancer. So far, results were presented in Table 3 in an “unstructured manner” with a variety of formatting errors and not informative due to the crude “listing” of studies. This table requires major revision and a harmonization of the different outcome variables, such as years of education or income, if possible, into low, medium, high SES. By the way, underreporting of the income is not just “forgetfulness”. This harmonized classification would allow a meta-analysis of the effect of SES by cancer site.

Discussion

There are excellent publications on cervical cancer in Latin America. Nation-wide HPV vaccination programs have been established in a many countries, for example also in Paraguay, with support of international agencies, including PAHO, covering all girls in schools. The next step is the detection of HPV infections and precancerous lesions (CIN) in young women. Here, SES may play a crucial role, but mainly with regard to the clinical treatment capacity of those tested positively with higher CIN stages, which is limited to metropolitan regions. Poorer women from rural regions cannot travel to distant health care centers. This problem is not captured when investigating the role of SES in CIN screening in rural regions.
