# Supplementary material for: Socioeconomic determinants of cancer screening utilisation in Latin America: A systematic review
Source: PLoS One. 2019 Nov 25;14(11):e0225667. doi: 10.1371/journal.pone.0225667 (PMC6876872; doi:10.1371/journal.pone.0225667)
Supplement: Attachment — Submitted filename: referee report.docx [file pone.0225667.s004.docx]

Bernardo Nuche-Berenguer and Dikaios Sakellariou present a well written manuscript; however some issues should be adequately addressed in order to improve the manuscript.

Method and extraction of this paper is not clear. Please add statements regarding:

-no. of publications in each language reviewed. In publications written in Spanish and Portuguese, were only abstract considered? or the authors extract the data from the body of the article? Add a flowchart describing the process of selection of publications in this section (Fig1).

Line 71- Among the socio-economic factors to search should be considered the distance of patients to urban areas, since in LA a large number of people live far from urban regions or in rural-areas?. As well as, data on ethnicity of patients I understand that unfortunately these data are not always available.

Line 89- Inclusion and exclusion criteria:

Authors should describe why the choice of these types of tumor (breast, cervical,

colorectal cancer) are most reported types of tumors in the region?

Line 94.- What is the reason to exclude the Caribbean islands?

Line 98- Data extraction:

Among the factors that can be included if the population lives in rural zones or located in cities?

Authors should describe if used a test statistic for OR? The confidence intervals should be placed in parentheses to separate them from the OR, in all cases.

Improve and use the same format for tables.

In the discussion, the authors mention that some LA countries have made progress in reducing cancer disparities, could provide concrete examples of the country and program, since depending on these recommendations can be extrapolated to other countries in the region. CicLAP and others grups?
